# Cyber-biological convergence: a systematic review and future outlook

**DOI:** 10.3389/fbioe.2024.1456354

**Published:** 2024-09-24

**Authors:** Mariam Elgabry, Shane Johnson

**Affiliations:** ^1^ DAWES Center for Future Crime at UCL, Jill Dando Institute for Security and Crime Science, London, United Kingdom; ^2^ Bronic, London, United Kingdom

**Keywords:** cyberbiosecurity, systematic review, engineered biology, policy, biosecurity, cyber threats, artificial intelligence, security

## Abstract

The introduction of the capability to “program” a biological system is referred to as Engineered biology and can be compared to the introduction of the internet and the capability of programming a computer. Engineered biology is supported by a digital infrastructure that includes data, data storage, computer-dependent laboratory equipment, internet-connected communication networks, and supply chains. This connectivity is important. It can improve workflows and enhance productivity. At the same time and unlike computer programs, biological systems introduce unique threats as they can self-assemble, self-repair, and self-replicate. The aim of this paper is to systematically review the cyber implications of engineered biology. This includes cyber-bio opportunities and threats as engineered biology continues to integrate into cyberspace. We used a systematic search methodology to review the academic literature, and supplemented this with a review of opensource materials and “grey” literature that is not disseminated by academic publishers. A comprehensive search of articles published in or after 2017 until the 21st of October 2022 found 52 studies that focus on implications of engineered biology to cyberspace. The search was conducted using search engines that index over 60 databases–databases that specifically cover the information security, and biology literatures, as well as the wider set of academic disciplines. Across these 52 articles, we identified a total of 7 cyber opportunities including automated bio-foundries and 4 cyber threats such as Artificial Intelligence misuse and biological dataset targeting. We highlight the 4 main types of cyberbiosecurity solutions identified in the literature and we suggest a total of 9 policy recommendations that can be utilized by various entities, including governments, to ensure that cyberbiosecurity remains frontline in a growing bioeconomy.

## 1 Introduction

Engineered biology is the design and fabrication of biological systems that do not already exist in the natural world into real world solutions^1^. Engineered biology is becoming more integrated within the cyber domain–as laboratories become more internet-connected and scientific research more computer-dependent (Mueller, 2021). For instance, the design of a microbial strain to express a desired protein (e.g., for medicinal use) relies on software and databases to generate the appropriate DNA sequence. These sequences are then transmitted digitally to a facility that will use this information to synthesize (produce) the new DNA molecules and cell lines that are grown in computer-controlled fermenters (Peccoud et al., 2018). At a time when broader communities are participating in developing engineered biology in unexpected ways, discussions of security are limited and confined to siloed expertise (Elgabry et al., 2022). Engineered biology may take advantage of the benefits of internet connectivity (e.g., exploiting cloud-based databases that store biological information) but not impact on cyberspace, or it may have a transformative impact on computing and the internet in the future (e.g., through DNA storage).

The objective of this review is to take stake of the current landscape and assess the cyber implications of engineered biology as engineered biology continues to integrate onto cyberspace. To do this, we apply a systematic search methodology to review the literature (e.g., Petticrew and Roberts, 2006) to address the following questions:

1. What are the main cyber opportunities,
2. and threats related to engineered biology?
3. What are the recommended solutions to the threats identified?
4. And where available, how quickly are these evolving (Boxes 1-6) and how will they transform cyberspace in the next 5–10 years?

### 1.1 Structure of this report

This report is organised thematically by research question. Section 2 details the review methodology employed. Section 3 summarises the identified opportunities (subsection 3.1) and threats (subsection 3.2) of engineered biology in cyberspace. Where estimates are available in the literature, we provide indications of how quickly these technologies are evolving (Boxes 1-6). Sub-section 3.3 summarises the recommended solutions to the threats identified in the literature. Section 4 synthesizes the recommendations and discusses possible routes to cyberbiosecurity.

The report concludes by providing focused policy recommendations, that can be utilized by various entities, including governments, to ensure that cyberbiosecurity remains frontline in a fast-developing bioeconomy.

## 2 Review methodology

### 2.1 Databases and search terms used

A search was conducted on 21 October 2022 to identify relevant articles for this review. We searched the academic electronic databases ProQuest Central^2^, ACM digital library^3^ and IEEE Xplore^4^. Collectively, these provide excellent coverage of published research across the social, engineering and physical sciences, as well as the information security literature. General web searches were also conducted to identify relevant reports and media coverage of known incidents of interest. While ProQuest Central indexes media reports, we conducted a general web search using Google (and Google News) Search to provide more extensive coverage. For robustness, we also used DuckDuckGo as another open search engine.

To search the above databases, the following search query^5^ was devised:

(“engineered biology” AND cyber) OR cyberbiosecurity

Over a series of iterations, we trialled different search terms to achieve an acceptable balance of relevant and irrelevant articles that would need to be sifted. We included the search term “cyberbiosecurity” as this an emerging discipline in which teams work to safeguard biological material, tools and systems integrated in the cyber domain. Moreover, these search terms, and not “synthetic biology”, were used to maintain a focus on tools affecting cyberspace as opposed to experimental research findings in the (wet) laboratory.

In addition to reviewing articles identified using the search engines, we employed “snowballing”, a method of retrieving additional relevant articles listed in the bibliographies of already identified articles.

### 2.2 Study inclusion and exclusion criteria

**FIGURE 1:**
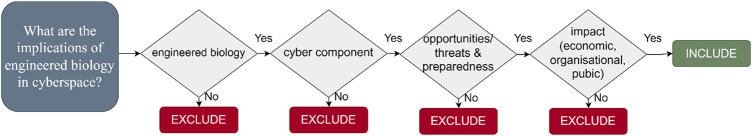
Decision tree used to identify articles for the review.

We devised a decision tree (see Figure 1) to determine if papers should be included in the review, and to ensure that the criteria (Table 1, organized according to the PICOS format (Richardson et al., 1995; Sackett et al., 1997; Schardt et al., 2007) were used consistently throughout the review (Byrt et al., 1993). Studies employing any methodology (e.g., qualitative and quantitative including systematic reviews and meta-analyses, Randomised Controlled Trials, cohort studies, case-control studies, cross-sectional surveys, case reports, position papers) were included.

**TABLE 1:** Summary of the eligibility criteria for the screening phases of the systematic review.

Criteria	Inclusion	Exclusion
Population(s)	Human, animal, environmental	All included
Intervention(s)	Current or future cyber opportunities, threats and recommended solutions to the threats identified related to engineered biology and/or cyberbiosecurity	All included
Comparator	Not applicable	Not applicable
Outcomes	Identified recommended solutions to the threats and possible routes to cyberbiosecurity	Opportunities, threats and recommended solutions only related to the integration of engineered biology with cyberspace (cyberbiosecurity)
Study design	All included	All included
Other	English language, open-source and/or accessible with institutional credentials	Non-English, paywall

All types of information sources were included with the exception of articles that were not available in English or that had to be purchased. Papers published before 2017 were excluded to ensure their contemporary relevance.

**FIGURE 2:**
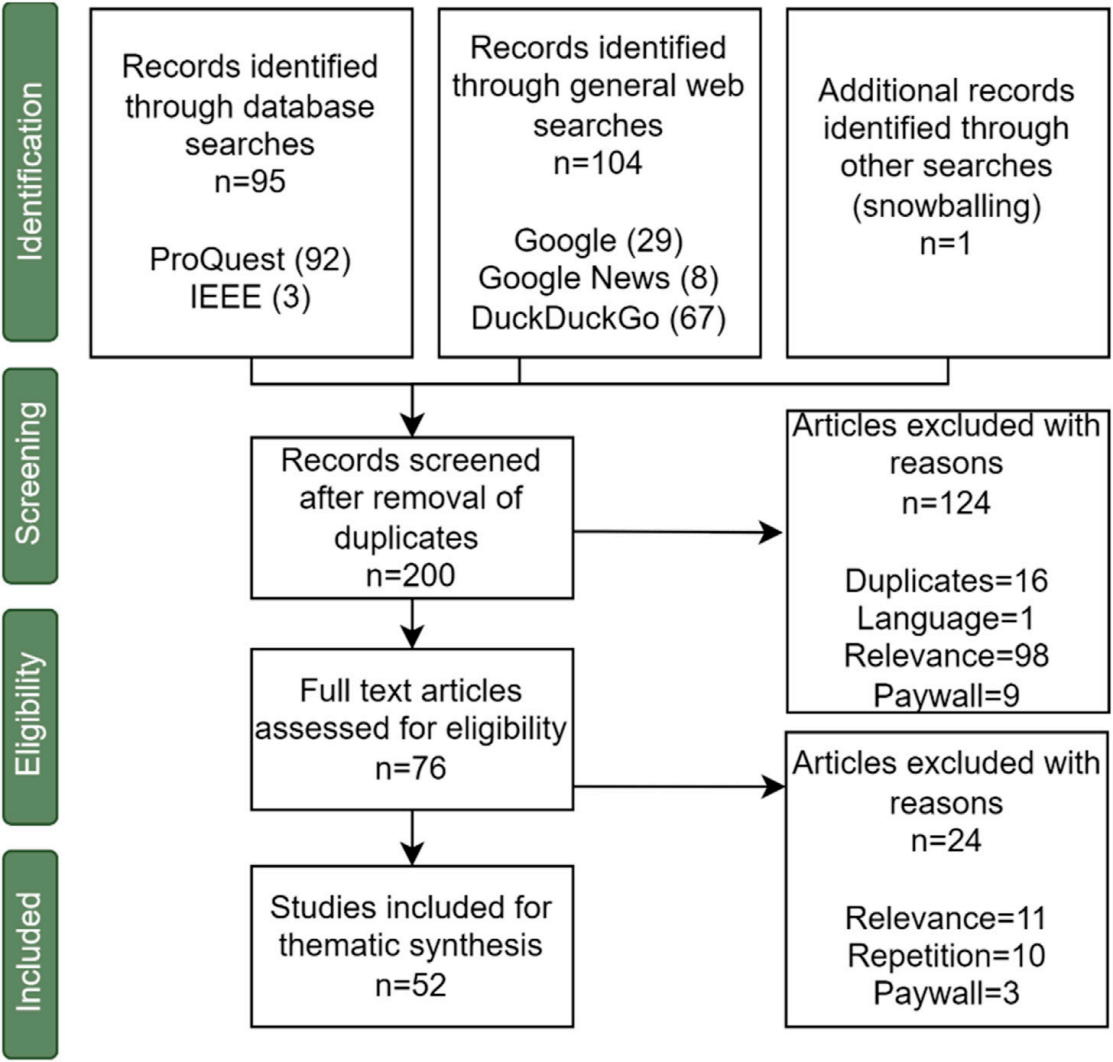
PRISMA chart of the number of articles identified, screened and ultimately reviewed.

For the academic review, EPPI Centre Reviewer (Thomas et al., 2010) was used to remove duplicates and for the screening and extraction of data. Figure 2 and Table 2 summarise the volume of articles identified (and excluded) at each stage of the search process using a Preferred Reporting Items for Systematic Reviews and Meta-Analyses (PRISMA)^6^ chart.

**TABLE 2:** Summary of the volume of articles identified (and excluded) at each stage of the search process.

Database/Method	Items	Duplicates	Total
ProQuest Central	92	13	79
IEEE Xplore	3	2	1
ACM digital library	0	0	0
Google Search Engine	29	1	28
Google News	8	0	8
DuckDuckGo Search Engine	67	0	67
Additional articles (e.g., through snowballing)	1	0	1
Total articles	200	16	184

In the first stage of screening, the titles and abstracts of the 200 articles identified were read and assessed against our inclusion criteria. For quality assurance, two researchers independently screened the titles and abstracts of 10% of the (200) identified papers and assessed whether they met the inclusion criteria. A metric called Inter-rater reliability^7^ was used to assess the alignment of the two coders in terms of whether they would include or exclude the articles whilst screening the titles and abstracts of the papers identified. There was perfect agreement for this exercise. Details of the databases searched, and from which the 200 articles were identified are shown in Table 2.

The full texts of the 76 articles that appeared to meet our inclusion criteria were then read and assessed against our inclusion criteria. Ultimately, 52 studies were found to meet the inclusion criteria, and for each of these articles, the following data were extracted:

• Year of study
• Publication type (journal, paper in conference proceedings etc.)
• Data analysed
• Study design (e.g., experimental study, focus group, interviews, Delphi method)
• Brief description of study
• Cyber opportunities identified
• Cyber threats identified
• Forecast timeframe and rate of development (i.e., for industry adoption)

A thematic analysis (Thomas and Harden, 2008) was subsequently used to synthesize and identify the impact of engineered biology in cyberspace and any recommendations provided in the literature.

### 2.3 Studies identified

**FIGURE 3:**
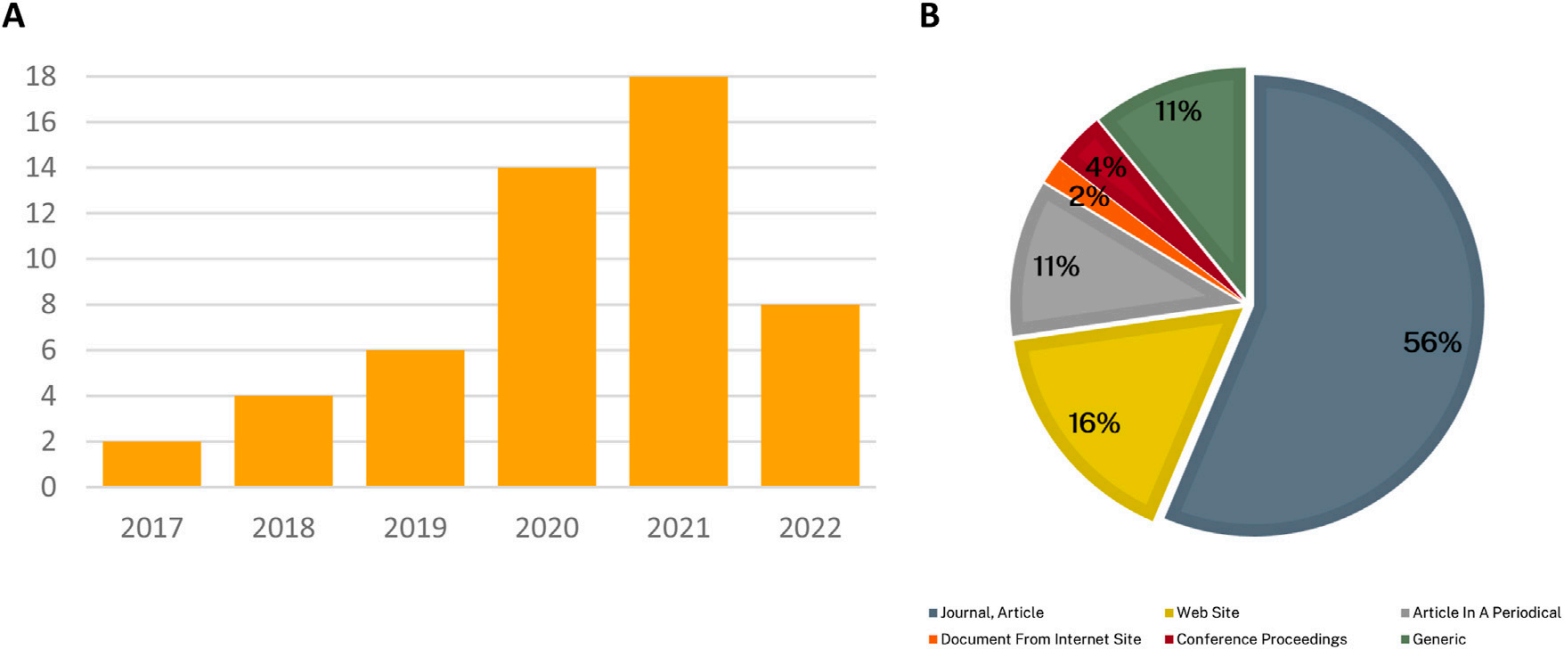
Descriptive results of included articles. Panel **(A)** Publication year of articles. Panel **(B)**. Article source types. (Articles in periodicals include magazines. Website sources include blogs and news articles found on the web. Conference proceedings are a collection of academic papers published in the context of an academic conference or workshop. Generic signifies other types of article sources such as pre-prints found in the bioRxiv database.)

Figure 3 shows the number of articles identified by year and publication sources. It indicates that the number of relevant articles identified in the search has increased year on year, and that the majority of articles were published in academic journals.

## 3 Results

### 3.1 What are the main cyber opportunities associated with engineered biology?

The science of engineered biology and its various applications from sustainability (Philp, 2021) to health (Li et al., 2021; Murch et al., 2018; Dixon, 2021) and the laboratory of the future (Reed and Dunaway, 2019; Mueller, 2021; Dixon, 2021) is accelerated by the rise of automation tools (Wintle et al., 2017; Richardson et al., 2019; Drape et al., 2021; Duncan et al., 2019; Dixon, 2021; Defranco et al., 2019; Boyle, 2020; Bartley et al., 2020; Gallup et al., 2021), bioinformatics (Li et al., 2021; Wintle et al., 2017), big data and digitisation (Murch et al., 2018; Richardson et al., 2019; Gallup et al., 2021; Dixon, 2021), biofoundaries (Philp, 2021; Dixon, 2021; Kitney et al., 2021) and convergence with other emerging technologies such as artificial intelligence (AI) (Benning et al., 2022; Dixon, 2021; Richardson et al., 2019; Boyle, 2020; Reed and Dunaway, 2019; Gallup et al., 2021; Bartoszewicz et al., 2021), Blockchain (Reed and Dunaway, 2019; Philp, 2021; Mohammadipanah and Sajedi, 2021; Kitney et al., 2021) and neurotechnology (Voigt, 2020; Murch et al., 2018; Dixon, 2021). In this section, we discuss the key cyber opportunities identified in the review and then consider how these may transform the future of cyberspace.

### 3.2 Bioinformatics, automation and bio-foundries will optimise biological design

Interpretation of biological data through computational tools of bioinformatics has led to the rapid development of engineered biology (Li et al., 2021). According to Wintle et al. (2017), Richardson et al. (2019) and Li et al. (2021), the rise of automated tools for biological design, test and optimisation free up the hands of laboratorians, allowing for more rapid and cheaper interrogation of a larger experimental landscape around the world. Such tools include computer-aided design (CAD) that aid the selection of parts and the design of genetic constructs (Gallup et al., 2021). The use of such tools make the production of novel materials, ultra-fast computers and chemical factories, easily programmable (Boyle, 2020). For parsing large datasets and optimising experimental design, other tools include data handling, debugging and statistical analysis software (Gallup et al., 2021).

Developments in engineered biology are being accelerated by the availability of big datasets, artificial intelligence (AI) and deep learning (DL), leading to the emergence of bio-foundries (Voigt, 2020). Bio-foundries, as numerous authors suggest, are facilities that employ AI-based software to automatically design and analyse experiments (Wintle et al., 2017), using liquid-handling robots in laboratories (Gallup et al., 2021; Defranco et al., 2019; Wintle et al., 2017; Richardson et al., 2019) that are instructed by this software with high accuracy and rapid throughput, to streamline complex experimental setups and improve reproducibility while also reducing biomanufacturing time. To illustrate, according to Philp, 2021, an automated laboratory in the UK was able to produce 17 potential molecules over a period of just 85 days, in some instances, with industrial-scale fermentations. A significant reduction of biomanufacturing time when compared to the typical lead time in biopharmaceutical products that are estimated to take 8–15 years^8^. According to Gallup et al. (2021), this automation of constructing DNA and the Design–Build–Test–Learn (DBTL) cycle can lead to “industrialisation” of engineered cells that can be tested in parallel (Boyle, 2020). Referred to as a “full stack” approach to engineered biology, Dixon (2021) gives examples in his article of companies that already provide services in biological design and bioinformatic software solutions like this, such as Benchling Inc. (who provide a cloud DNA sequence design and analysis platform), Synthace ltd. (who provide laboratory experimental automation), TeselaGen Biotechnology, Inc. (who have developed an AI-powered drug-discovery platform) and Synthego corp. (who offer a machine learning (ML) aided gene editing platform).

Bio-foundries deploy biomanufacturing workflows that are high-throughput, automated and modular (Dixon, 2021). According to Philp, 2021, bio-foundries were initially tested by the Massachusetts Institute of Technology (MIT), who were tasked to build organisms that can produce 10 molecules within 3 months. The MIT-Broad Institute bio-foundry succeeded in producing 6/10 targets. In the UK, according to there are five bio-foundries: Genome Foundry (Edinburgh), IBioIC (Glasgow), SynbiCITE (London), Earlham Biofoundry (Norwich), and SYNBIOCHEM Biofoundry (Manchester). Outside of the UK and as highlighted, Ginkgo Bioworks Inc. is considered an example company at the forefront of having a bio-foundry or otherwise referred to as “cloud lab” creating AI–enabled workflows. Ginkgko Bioworks Inc. also offers a proprietary bio-design platform to new start-ups through its venture capital programme (Gallup et al., 2021; Dixon, 2021). The authors also mention Microsoft Inc.'s Station B in the UK, which focused on building a platform for “programmable biology” through its cloud lab. However, this project has since been retired.

Streamlined biomanufacturing has several benefits to include vaccine production to precision agriculture (Kitney et al., 2021; Drape et al., 2021; Duncan et al., 2019). Regarding vaccine production, the current mass production paradigm is centralised and involves physical transfer of temperature-dependent vaccines. According to Kitney et al. (2021), this contrasts with bio-foundries that could be distributed small-scale manufacturing sites in many locations communicating digitally and applying Design–Build–Test–Learn (DBTL) cycle operations closer to the point of care. Regarding agriculture, the adoption of data-driven technologies helps the industry meet the growing food and sustainability demands through farm-to-table food production, processing and distribution systems (Drape et al., 2021; Duncan et al., 2019; Dixon, 2021; Murch et al., 2018). According to Dixon (2021) and Duncan et al. (2019), data generated from both applications are becoming an increasingly essential component to scaling up the engineering of biology, both in agricultural and the pharmaceutical industry and hence they provide a national advantage for nations that have access to such data. According to Dixon (2021), the potential of the bioeconomy and the emerging circular economy^9^ may soon be realised through the marriage of bio-foundries and “bio-informational engineering” platforms (see Box 1). Briefly, precision agriculture could, in the future, be enabled by “sentinel” plants that communicate to smart farms through the “Internet-of-Biological-Things”. Such integrated systems could realise economically competitive carbon-neutral and carbon-negative manufacturing processes.

BOX 1 **Bio-informational engineering as proposed by Dixon (2021).**

The *Internet-of-Biological-Things* is a network of connected biosensors and biological devices distributed across geographies for real-time and constant information exchange. This could enable precision agriculture with next-generation robotic architectures.

*Precision agriculture* refers to smart farms with plant-enabled sensing and satellite architecture including edge-computing^10^ with low latency, that leverages artificial intelligence, various datasets such as weather forecasts and geospatial insights to provide autonomous productivity and route planning of fertilisation through the use of drones.

In their articles, Boyle (2020) and Richardson et al. (2019) identify drug design and development, genomics, and (see also Gallup et al., 2021) protein folding as further applications for the use of artificial intelligence (AI) to extend current applications of engineered biology. Gallup et al. (2021) specifically stress the role that deep learning (DL) (a form of AI) could play in the analysis of microscopy images to help predict protein structure (e.g., protein structure prediction by the company AlphaFold) and inform novel drug discovery in the form of new antibiotics. In the future, according to Gallup et al. (2021), DNA design will be optimised using DL that would allow the optimal “writing” of DNA sequences for certain combinations of genetic parts and genetic contexts simply based on high-level commands–like programming a computer to execute a function. Other applications of AI for “*biology as an information science*” (Wintle et al., 2017; Li et al., 2021; Benning et al., 2022; Dixon, 2021; Richardson et al., 2019; Boyle, 2020; Reed and Dunaway, 2019; Gallup et al., 2021; Bartoszewicz et al., 2021) include a DL model developed by Bartoszewicz et al. (2021) that can predict if viruses such as Ebola and SARS-CoV-2 can infect humans directly from input data of sequenced DNA of the virus.

At the same time, ‘omics’^11^ data on cells, genes, transcripts and proteins continue to grow exponentially each year (Gallup et al., 2021). To make genome engineering for the gigabase scale (i.e. 1 billion bases) possible, according to Bartley et al. (2020), engineered biology needs automation languages, workflows and graphical representations in place. According to Gallup et al. (2021), this could change the whole process of design in synthetic biology (see Box 2), shifting current practice of gene circuit design before experimentally testing host cells to computationally testing directly within *in silico* whole-cell simulations. The authors provide the example of the fully-sequenced bacteria *M. genitalium* (∼500 total genes), which is already computationally demanding in exploiting such data to make a simulation of how all the genes and proteins behave. However, as gene numbers increase (e.g., 1,000+ for *E. coli*) it may not be possible to run a single simulation with the current limits to computational power (Box 2).

BOX 2 **How quickly are engineered biology technologies evolving and how will they transform cyberspace in the next 5–10 years?**

Estimating exactly when technologies will be deployable is difficult, but for most technologies progress is exponential and not linear (Farmer and Lafond, 2016). Below we summarise predictions extracted from the systematic search.

**Today**

Engineered biology products already on the market generate around $2 billion in worldwide annual sales (Voigt, 2020). Examples include genome edited livestock of which more than 67 examples exist, such as hornless cattle (eliminating physical dehorning), sheep with longer wool, goats that make milk with human whey protein, virus-resistant pigs, and chickens that lay allergen-free eggs.

**5–10 years**

According to Voigt 2020, by 2030 it is highly likely that everyone will have either eaten, worn or been treated by at least one engineered biology product. Dixon (2021) predicts that in the next decade, it is likely that the integration of biological informational inputs will lead to a range of human-designed living monitoring systems that could realize microbiological-level surveillance networks that communicate in real time via satellite link for novel intelligence collection, the monitoring of emerging infectious diseases and the automation of agriculture.

**>10 years**

Bartley et al. (2020) suggest that by around 2050, the infrastructure to support gigabase genome processing will require a team with the capabilities of around 500 investigators catalysed by two technologies advancing faster than Moore’s law: DNA sequencing and synthesis. To realise this, according to the authors, technical support for the integration of modelling and design at the gigabase scale is needed as is a better understanding of the relationship between genotypes and phenotypes.

Dixon (2021), Voigt (2020) and Gallup et al. (2021) describe a future of engineered biology where the biological and information sciences will become increasingly more difficult to define and “*where cells are designed to work together or be integrated into non-living materials or electronics*”. Voigt (2020) predicts that products will shift to systems, Gallup et al. (2021) state that a “‘*cells as modules’ approach could offer plug-and-play organisms and consortia that can be thrown into new systems, like reusable functions in computer code*.” The “auto-streamlined” genomes could be used for directed evolution dynamic synthetic genomes that in the near future, could engineer themselves for specific fixed tasks as their cells differentiate. For example, they may delete regions and genes no longer required for their roles autonomously. Achieving a synthetic or artificial cell will help us create self-replicating entities or systems that can compute, self-assemble, and 1 day be able to achieve autonomous self-replication (Gallup et al., 2021). Current projects pursuing this include Build-a-Cell (United States) https://www.buildacell.org/and SynCellEU (Europe) https://www.syntheticcell.eu/.

Some argue that cyber opportunities for the new era of engineered biology require data infrastructure that can support it (see Box 3) with standardised data exchange formats, data management and curation methods, metadata reporting, and data interoperability using open-source software (Gallup et al., 2021). With these in place, data sharing between research organisations, companies and other bodies can be facilitated to accelerate advancements in engineered biology that could also lead to autonomous multi-scale bio-foundries (see Dixon, 2021). A point to consider is that this model may challenge the traditional institutional life science practice of centralised experimentation and benchwork by researchers (Gallup et al., 2021). According to Gallup et al. (2021), classic science may need to keep pace and adopt “academic bio-foundries” that enable lab work via the cloud, and to collaborate more with industry for a more efficient way of working in the future (see Box 4 – lab of the future). Dixon (2021) and Reed and Dunaway (2019) look further into the future of AI-enabled engineered biology and imagine automated decisions and generated insights based on Big Data pattern analysis inside and outside of the laboratory. The avoidance of error, incidents, and accidents within and across laboratories may be enabled by AI for every networked laboratory “node”. Within a laboratory, according to Dixon (2021), the human effort of iterating through generations of designs will no longer be needed as ML models will reduce the time required to design a biological solution for a given problem set.

BOX 3 **What technical barriers are affecting how quickly engineered biology will evolve in the next 5–10 years?**

**Today**

Technical challenges remain to efficiently transfer biological innovations from the laboratory to the industrial scale (Gallup et al., 2021). Bartley et al. (2020) further highlight that two critical challenges need to be addressed: accessing well-annotated source genomes and representing/exchanging designs for modified genomes.

Over the period 2002–2010 the cost of DNA sequencing decreased from $3 billion to US$50,000 by 2010 and in 2014, the company Illumina offered human genome sequencing for US$1,000 (Dixon, 2021). In 2020, BGI (formerly known as the Beijing Genomics Institute) announced it would soon be able to offer the sequencing of a human genome for US$100 (Dixon, 2021). Cunningham and Geis (2020) note that DNA synthesis cost have changed more slowly than sequencing and editing costs. And that this is because the cost of nucleotide precursors and reagents have stayed essentially the same over the past decade. Nevertheless, the cost has decreased with Dixon (2021) highlighting that in 1980 the DNA synthesis of approximately ten nucleotides cost US$6,000, but by 2010 the synthesis of a million 60-nucleotide oligos cost just US$500.

**5–10 years**

Cunningham and Geis (2020) state that recreating smallpox in a private lab today costs around $3 million but that a similar effort in 2025 may cost as little as $100,000. These costs are perhaps the reason why there have only been small number of engineered biology commercialisations to date (Li et al., 2021; Voigt, 2020; Philp, 2021). Voigt (2020) provides the example of the anti-malarial Artimisin which was taken into production by Sanofi. However, this was discontinued because the cost synthesizing it was ultimately higher than sourcing it from plants.

BOX 4 **Converging technologies for the laboratory of the future (Reed and Dunaway, 2019; Mueller, 2021; Dixon, 2021; Mohammadipanah and Sajedi, 2021)**

Virtual personal assistants such as smart speakers, voice assistants, devices connected via Bluetooth and electronic laboratory notebooks can help realise the “*laboratory of the future*.” For example,:

• In contrast to physical laboratory notes, Electronic laboratory notebooks enable more secure data collection, storage and processing when encrypted, password-protected and stored on the cloud. Electronic laboratory notebooks can enable an audit trail with e-signature features that can enhance laboratory quality management, compliance and Good Laboratory/Manufacturing Practices.
• Voice-activated equipment can be used as a prevention mechanism for work surface contamination (due to decreased touch) while also increasing the efficiency and productivity of laboratory staff.
• Biometric authentication and blockchain technology^12^ can improve security in the laboratory.
• Wearable monitoring systems can track health in high containment laboratories to prevent errors, incidents and accidents.
• Virtual reality can be used for training laboratory staff.

### 3.3 Other opportunities: The future of bio-electronics, optogenetics and DNA storage may transform cyberspace

#### 3.3.1 Bio-electronics

Cyber opportunities also lie in the future of bio-electronics (Voigt, 2020; Dixon, 2021; Philp, 2021) thanks to the implementation of converging technologies such as robotics, parallel strain engineering^13^ and AI (Wintle et al., 2017; Li et al., 2021; Benning et al., 2022; Dixon, 2021; Richardson et al., 2019; Boyle, 2020; Reed and Dunaway, 2019; Gallup et al., 2021; Bartoszewicz et al., 2021). According to Voigt (2020), Zymergen, a ML-aided genomics company^14^, have made hyaline, a thin film from engineered organisms that can be used for flexible electronics in, for example, wearable technology and/or foldable smartphones. The electronic properties of particular materials and/or organisms can be further adjusted by engineering the nanostructure to make ultralight batteries, catalysts, solar cells, and optics. As an example, Voigt (2020) discusses melanin (the substance that in humans produces hair, eye and skin pigmentation). Depending on its structure, melanin can be a UV protectant (in humans), a photovoltaic cell (in wasps) or a luminescing pigment (in birds), and can be used to make ultralight batteries.

#### 3.3.2 Optogenetics

Optogenetics is a technique that enables the precise control of cells through gene expression in response to specific wavelengths of light (Dixon, 2021). According to Dixon, it introduces a novel electrical and electrochemical approach to control cell behaviours with various potential applications, including smart-phone-mediated insulin release in mammalian cells, and light-activated and adjusted plants in greenhouses. Current research organisations that already use this technique (see Dixon, 2021) include the company Berkeley Lights, which uses light to move individual cells (e.g., antibodies) automatically into individual, nanolitre-sized chambers to streamline research experiments.

#### 3.3.3 Engineered living materials

Engineered biology as a manufacturing discipline could lead to the development of advanced materials (Gallup et al., 2021). According to Gallup et al. (2021), engineered living materials (ELMs) can offer sustainable textiles and building materials using (say) bacterial cellulose, mushrooms and spider silk, some of which can already be found in the market (see Background subsection 1.2 and e.g., Balenciaga’s mycelium-based coat^15^). Such materials could be combined with optogenetics to enable cells to act as deployable miniaturised material “factories” that can be modified at a molecular level; a complex task for conventional machines, yet for microbes this is innate and at no added cost. Gallup et al. (2021) provide the example of an existing gel-like material containing heart cells that has been engineered to be light-sensitive so that it beats in response to a light pulse in muscle-like soft robots.

Future applications include the integration of electronics and engineered living cells to enable robots and brain-computer-interfaces (BCIs) to generate energy from the environment or for navigation (Voigt, 2020). In their reviews, Murch et al. (2018) and Dixon (2021) discuss neuromorphic^16^ devices for biological interfacing instead of using inorganic alternatives. Such devices may take the form of an optogenetic implant integrated with an electroencephalography (EEG)-based wearable to view health information. This, according to the authors, could lead to the 3D printing of personalised genomics, medical and fitness devices for human *in situ* use such as integrated wearable and smartphone technologies to allow for (say) the controlled release of chemicals in the gut to regulate health.

#### 3.3.4 DNA storage

Engineered biology can further transform cyberspace by introducing the capacity for biological material such as DNA for information storage (Philp, 2021). Global internet traffic and the associated electricity needs for the information technology sector has grown significantly, increasing 12-fold since 2010. In Philp, 2021 review, it is noted that all of the current information on the internet could be stored in only 1 g of DNA. DNA storage could offer a “low maintenance and low energy system” to store the entire world’s data once the costs of achieving this are reduced.

Unfortunately, the costs of these technologies and the expected timelines were not explicitly discussed in the literature reviewed.

### 3.4 What are the main cyber threats associated with engineered biology?

In parallel to the enormous benefits engineered biology has to offer in isolation and in concert with other technologies (e.g., AI), there are concerns of dual-use. In fact, researchers have noted that synthetic biology is a “double-edged sword” (Li et al., 2021) and is “inherently dual use” (Cunningham and Geis, 2020). In this section, we discuss the key threats identified in the review and then consider the crime opportunities that these create.

#### 3.4.1 Artificial intelligence misuse and biological dataset targeting

Authors have discussed the risk and dangers of actors maliciously automating the manipulation of medical datasets (Pauwels, 2020; Richardson et al., 2019; Palmer et al., 2021; Mantle et al., 2019; Murch et al., 2018). Two examples provided by Pauwels (2020) include a malicious attack designed and tested by researchers from Cornell University in 2018, that targets lung CT scans in hospitals to generate false indications of tumours. This led to a misdiagnosis rate of over 90%. The second example, demonstrated by Harvard researchers (Pauwels (2020), involved minor alterations of skin cancer images in biopsy results that corrupted the diagnosis.

Biological information such as DNA, according to Rizkallah (2018), is the “*ultimate personal identifying information*”. It is unique and irreplaceable which makes it highly valuable. According to researchers (Cunningham and Geis, 2020; Dixon, 2021), enough individuals in the US have completed commercial genetic tests and have publicly shared their genetic information, that 90% of individuals of EU-US descent are identifiable through their DNA. This is a severe concern particularly for spies, soldiers, and their families who are vulnerable to threats, attacks, or exploitation through espionage or from publicly available data sources. Lanier (2018) and Reed and Dunaway, 2019 point out that this digitised data (e.g., DNA) is usually stored by university researchers on computers, local area networks and/or cloud services that transfer the (often unencrypted) data between users over email or other (unsecure) sharing technologies, making it vulnerable.

#### 3.4.2 Targeting and hacking of insecure internet-of-medical-things

Medical devices are increasingly internet connected. While this increases functionality, it creates opportunities for cyber-attacks that have the propensity to cause direct harm to human health (Potter and Palmer, 2021; Rizkallah, 2018; Logstail, 2022; Reed and Dunaway, 2019; Dixon, 2021; Cunningham and Geis, 2020). For example, Potter and Palmer 2021 note that smart watches record activity about an individual’s lifestyle that a malicious actor could intercept (e.g., via public Wi-Fi or Bluetooth, see Logstail, 2022) and exploit. In more extreme examples, researchers (Logstail, 2022; Reed and Dunaway, 2019) describe incidents in which a pacemaker was comprised to produce a lethal voltage shock, and an insulin pump manipulated to deliver a fatal dose of insulin to the wearer. According to Reed and Dunaway (2019), this knowledge led to the deactivation of the (un)secure wireless connection of Vice President Dick Cheney’s defibrillator in 2013 to prevent the possibility of it being remotely inactivated.

Logstail (2022) further identified threats associated with human-implanted devices that could be targeted and exploited. For example, the article suggests that eye/hearing implants could be exploited using a man-in-the-middle attack^17^, and that blood test implants could be hacked via an SQL injection attack^18^ to retrieve critical data. The article mentions other attack methods that can be used to steal data from such devices, and to change their settings, including turning them off. Finally, and perhaps equally concerning, the article highlights the extension of the attack surface into connected networks such as hospitals to which the devices connect and exchange data.

#### 3.4.3 Other data and device risks

Additional risks to data and devices emerge from the following issues: the under-reporting of incidents which limits our understanding of the risks (Gertner, 2021), a lack of wargame activity to identify and address vulnerabilities for bioprocessing teams (Potter and Palmer, 2021), less tacit knowledge in the Life sciences (Mueller, 2021), naïve trust in the biotechnology research industry (Peccoud et al., 2018; Mueller, 2021), gaps in (security) expertise (Mueller, 2021), a misconception that IT suffices to address threats, and incomplete awareness by life scientists on the potential threats (Mueller, 2021). These issues will now be discussed in more detail.

A New York times reporter (Gertner, 2021) interviewed biosecurity experts about the highest biosecurity level (BSL-4) laboratories and their current security measures. According to one expert interviewed, no official international database exists to keep track of these types of labs and there is no requirement for governments to acknowledge their existence. Another biosecurity consulting company interviewed by the reporter mentioned that incidents and exposures do occur but that there is no reporting of them. Peccould et al. (2018) and Mueller (2021) identify the risk of a “naïve trust” in the biotechnology industry and amongst life scientists–noting that they will share data and biological samples without first establishing the intended use, as well as trust that digital sequences match the physical sequences shared. Mueller (2021) further comments on the “incomplete awareness” in the life sciences noting that researchers are “*mostly ignorant of the dangers as they are barely trained in security issues*” (p. 12) if at all. This absence of expertise regarding cyber-biorisks is what Mueller (2021) describes as “*less tacit knowledge*” or the “*misconception that life sciences are shielded from malicious interventions”* (p. 12) because research requires specific expertise and technical skills through constant practice and peer observation. According to Potter and Palmer 2021, the lack of wargame activity or an adversarial approach (Elgabry et al., 2020) to identifying potential security exploitations also contributes to the challenge of identifying and addressing these threats which can lead to opportunities for crime.

#### 3.4.4 The crime opportunity landscape

The expansion of the bioeconomy through engineered biology inevitably creates greater opportunities for crime (Philp, 2021). Elgabry et al. (2020) conducted a systematic review of these threats. In that study, 794 articles were initially identified, of which 15 were ultimately relevant to the review (research describing a threat model facilitated by synthetic biology). Across these studies, eight potential crime types were identified that could be expected to emerge within the next 5–10 years. These were: bio-discrimination, cyber-biocrime, bio-malware, illegal biohacking, at-home drug manufacturing, illegal gene editing, genetic blackmail, and illegal neuro-hacking. Each of these are now discussed.

##### 3.4.4.1 Genetic Blackmail

Genetic Blackmail is the misuse of DNA information for extortion (Elgabry et al., 2020; Dixon, 2021; Logstail, 2022; Lanier, 2018). Lanier (2018) points to research conducted by Israeli scientists who showed that it is possible to fabricate DNA evidence (blood and saliva samples) to match a person other than that of the donor (e.g., DNA from a parole database) without obtaining any tissue from that person. This may enable the planting of “spoofed” DNA at crime scenes to misdirect law enforcement (Dixon, 2021; Elgabry et al., 2020).

##### 3.4.4.2 Bio-discrimination

Bio-discrimination and socio-genomics are defined as the discrimination against, or targeted extortion of, individuals and/or groups based on their genotypes, phenotypes, and/or behaviours–turning databases with health information (e.g., health records, insurance profiles) into cyber-targets which can also be monetised in several ways (Elgabry et al., 2020; Jordan et al., 2020; Defranco et al., 2019). According to Dixon (2021), advances in social genomics may lead to the illicit acquisition of genomic information and medical data as a pathway for intelligence operations such as grey-zone warfare. Traits such as loyalty and addictiveness may soon be correlated with genetic and epigenetic^19^ patterns harvested from a target’s biological profile which could be exploited by intelligent agencies’ when recruiting staff. Genetic data can also be lucrative as it could be sold to insurance companies (see Lanier, 2018; Potter and Palmer, 2021; Jordan et al., 2020) or to athletes interested in masking their own genetic conditions (Lanier, 2018), in the same manner that they may be currently masking a drug test, or for targeted advertising of vulnerable patients (Jordan et al., 2020).

##### 3.4.4.3 Hacking for ransom

Researchers (Palmer et al., 2021; Murch et al., 2018) have identified hospitals and medical devices (e.g., insulin pumps) – which are prone to hacking–as ransom targets. For example, Lanier (2018) discusses an Indiana hospital that had to pay $55,000 to hackers in 2018. Philp (2021) notes that cyberattacks will increase the more biology becomes digitised. In addition to hospitals, Duncan et al. (2019) estimate that more than 20% of small agribusiness (<100 employees) in biotechnology are hacked as employees and companies lack relevant policies for basic cyber hygiene (e.g., personnel using personal computers for business activities), which increases the risk of cyber-attacks.

Authors highlight how hackers could disrupt the biomanufacturing of important medicines for human health by hacking internet connected freezers, refrigerators and incubators (Dieulis 2020; Reed and Dunaway, 2019; Mueller, 2021), or manipulate thermal processing time and temperature to compromise food safety (Duncan et al., 2019). Lanier (2018) suggest that vulnerabilities exist in systems that hackers could exploit to compromise a device with the intention of stalling the production of critical drugs. For example, according to Duncan et al. (2019) this could be achieved by using a computer worm to seize control of robots or autonomous vehicles leading to the failure to perform and overriding precise function of such devices.

##### 3.4.4.4 Corporate espionage

Corporate espionage was mentioned as a crime type in 11 articles (Jordan et al., 2020; Reed and Dunaway, 2019; Rizkallah, 2018; Mueller, 2021; Dieulis 2020; Peccoud et al., 2018; Millet et al., 2019; Duncan et al., 2019; Potter and Palmer, 2021; Elgabry et al., 2020; Lanier, 2018). Several authors (Reed and Dunaway, 2019; Rizkallah, 2018; Mueller, 2021) highlight how the penetration of corporate networked laboratories can allow a malicious actor to steal intellectual property or an organization’s sensitive scientific and business data. Doing so can halt company operations entirely or offenders may threaten to revoke access, seeking millions of dollars in ransom (Lanier, 2018; Millet et al., 2019). As a consequence of such attacks, an organisation’s reputation could be severely affected (Reed and Dunaway, 2019; Mueller, 2021) challenging its viability. Another risk concerns insider threats, whereby rogue actors (which can include state actors) inside a laboratory steal information for monetary or other gains (Elgabry et al., 2020).

#### 3.4.5 Emerging crime forms

##### 3.4.5.1 Bio-malware and neuro-hacking

Emerging concerns (Elgabry et al., 2020; Mueller, 2021; Palmer et al., 2021; Dixon, 2021) that do not neatly fit standard biosecurity or cybersecurity threats were also identified (Box 5). To illustrate, several authors (Elgabry et al., 2020; Farbiash and Puzis, 2020; Peccould et al., 2018; Reed and Dunaway, 2019; Cunningham and Geis, 2020; Murch et al., 2018; Lanier, 2018; Palmer et al., 2022) discuss the use of biological malicious software (or bio-malware). That is, a DNA-based attack where computer malware is inserted into physical genetic material that when sequenced compromises the computer, providing an offender with remote access to it. According to Palmer et al. (2021), although interception via typical electronic formats such as phishing may be easier and quicker to perform, the covertness of bio-malware may become an attractive attack method for adversaries, or could become a means of smuggling information (digital and/or biological) across borders (Potter and Palmer, 2021).

BOX 5 **How quickly is engineered biology evolving and how will it transform cyberspace in the next 5–10 years?**

**Today**

According to the Cunningham and Geis (2020) engineered biology is enabling “Biohacking” (tinkering with biology). The authors highlight that biohacking became a major trend on the Gartner Hype Cycle as an emerging transformative technology in 2018, and has since become mainstream. Any member of the public can today use open-source bioinformatics tools and databases, as well as purchase kits online for “Do-It-Yourself Bacterial Gene Engineering CRISPR editing” for just $169. The annual MIT-founded and sponsored synthetic biology competition “International Genetically Engineered Machine (iGEM)” features 6,000 competitors from high school, college, and private industry seeking to produce the best synthetic biology designs using these open-source tools. The authors highlight an example project in 2018 of an undergraduate team building “*Printeria, a fully equipped bioengineering device able to automate the process of printing genetic circuits in bacteria*”.

**5–10 years**

Defranco et al., 2019, Potter and Palmer 2021, Elgabry et al. (2020) all highlight the emerging crime trends of engineered biology, predicting that within the next 5–10 years ongoing developments in neuroscience (Defranco et al., 2019) and biotechnology (Potter and Palmer, 2021) will grow in value for operational use in bio-discrimination, bio-malware, biohacking, cyberbiocrime, warfare, intelligence, and national security (WINS) applications.

Future crimes according to Elgabry et al. (2020) include illegal gene editing, DIY drugs, genetic blackmail, and neuro-hacking.

Neuro-hacking was also identified as an emerging method of offending. It is described as the covert manipulation of “gut-therapies” used by individuals by a malicious actor (Wintle et al., 2017). Forms of “gut-therapies” already exist commercially in the form of probiotics and prebiotics. These are intended to induce a “healthy” balance of the gut microbiome^20^ but could, in the future, be exploited for malicious purposes through neuro-hacking. According to Defranco et al., 2019, this could be achieved through the use of neuro-data (retrieved from genomic data, devices or neurotechnology) to cause harm to an individual or group directly/indirectly or to engineer a particular effect (e.g., change of mood, behaviour) in an individual or a group. “Gut-therapies” can be ingested or implanted in the intestines, or other locations of the human body to collect health-related data to support the development of novel therapeutics and diagnostics (i.e., theranostics) (Bernal et al., 2020; Elgabry et al., 2020; Wintle et al., 2017). According to Bernal et al. (2020), in the future “Gut-therapy” theranostics will comprise of biosensors built from engineered bacterial populations that can be remotely monitored using conventional network infrastructure. Engineered bacteria can be controlled through external electric signals, however, due to the resource-constrained nature of engineered cells, security mechanisms to avoid or prevent malicious stimuli cannot be implemented (Bernal et al., 2020). Bacteria have natural defence mechanisms, such as the production of biofilms that could be targeted and “hijacked” as one form of attack. Bernal et al. (2020) demonstrate such a cyberbioattack whereby a (biological) distributed denial of service (DDoS) attack was used to affect a bacteria-based biosensing system using malicious jamming signals (a series of coordinated emission of molecular signals) to disrupt the generation of biofilms.

##### 3.4.5.2 Cyber-biocrime

Collectively, these threats may introduce new types of crimes that are not yet legislated for (Elgabry et al., 2020; Richardson et al., 2019, and Ibrahim et al., 2020; Farbiashand Puzis, 2020; Reed and Dunaway, 2019). Cyberbiocrime is defined as “*criminal activities carried out by combined means of computers/Internet and biological/biochemical material*” (Elgabry et al., 2020). In the Elgabry et al. (2020) systematic review, 46% of the identified crime exploits were “Biotechnology-dependent”, of which more than 30% were cyber-related. Biotechnology-dependent offences are those that cannot be committed without the use of biotechnology, while Biotechnology-enabled crimes (54% of the identified crime exploits) are traditional offences that are in some way extended in scope by biotechnology (Elgabry et al., 2020).

Unlike computer software, biological systems cannot be “patched” once released in the wild, nor are there easy ways to “patch” the humans (or animals or crops) susceptible to such agents (apart from pursuing biocontainment) (Schneier and Larisa, 2019). Biological material and DNA sequences could be intercepted and manipulated (maliciously) to produce pathogenic self-replicating entities, for example, (see Mantle, 2019; Cunningham and Geis, 2020). Mueller (2021) highlights that bio-foundries may unintentionally and unknowingly receive customer digital information that will result in the production of harmful components of biological agents. Opportunities for such exploitation include devices that are increasingly connected and automated, such as PCR machines (see, Richardson et al., 2019). Recent studies suggest that these opportunities raise unprecedented security concerns, creating a whole new category of potential weaknesses labelled “cyberbiosecurity threats” (Ibrahim et al., 2020). In fact, Farbiash and Puzis (2020) describe the weaknesses in the current Screening Framework Guidance for Providers of Synthetic Double-Stranded DNA, which is intended to prevent unauthorised access to biological materials that would be of concern (e.g., toxins). The authors showed that they could circumvent the screening protocol used to prevent this using malware (a generic obfuscation procedure) through a malicious browser plugin within a biological lab. This tricked a biologist into producing a substance of the attacker’s choice. The scenario the authors demonstrated showed that relying only on standard end-to-end encryption provided by HTTPS does not help when data may be corrupted. That is, adversary-resilient biological protocols are critically needed.

Currently, the gene libraries most commonly used by researchers (e.g., GeneBank, NCBI) do not provide digital signatures of acceptable submissions for data downloaded and do not require orders to be validated. This means that gene libraries are unable to employ intrusion detection approaches to identify malicious code. On the biological side, Mueller (2021) describes “active biologicals” which can raise unique concerns if an undeclared (and ‘invisible’ dangerous) biological component (protein or nucleic acid) in a formulation is released from the packaging of the product or in the retail chain. Reed and Dunaway, 2019 describes cyber-biosafety issues whereby a malicious actor may (for example,) alter electronic genomic sequences to enhance how infectious or drug resistant a microorganism is, or the range of hosts it can affect. According to Reed and Dunaway, 2019 potential exposure to such microorganisms can then be achieved by the adjustment of fan speeds in laboratory building ventilation systems to alter pressure differentials between administrative and laboratory workspaces, leading to the release of the pathogen.

#### 3.4.6 National security threats and targets

Infrastructures targeted by malicious actors could range from vaccine production sites to critical food and agriculture farms (Kitney et al., 2021; Reed and Dunaway, 2019; Dotmatics, 2022; Palmer et al., 2022; Dixon, 2021; Mueller, 2021; VT, 2022; Richardson et al., 2019; Millet et al., 2019; Drape et al., 2021) leading to the potential for industrial espionage (Palmer et al., 2021) with national security implications (Townsend-Drake et al., 2021; Palmer et al., 2021; Palmer et al., 2022; Dixon, 2021; Jordan et al., 2020; Millet et al., 2019; Mantle et al., 2019; Cunningham and Geis, 2020; Richardson et al., 2019). For example, a ransomware attack could severely impact the supply chain of the US. meat industry, delaying production that can influence distribution and the availability of meat to retail consumer outlets (e.g., grocery stores, restaurant chains, large food distribution companies) and food companies relying on these sources as ingredients (Virginia Tech., 2021). According to Drape et al., 2021, cyber-attacks to agriculture are underreported due to the lack of detection capability despite the food industry relying on computer systems. Duncan et al. (2019) highlight how the food and agriculture system is highly susceptible to sabotage. They discuss how military food production, including soldier meals, can be compromised with little to no manufacturing know-how by merely lowering the temperature of meat cookers before packaging, for example,. Other high-value food and agricultural products susceptible to cyber threats include “*high-yielding and specialty agricultural crops, high performance livestock…[and] biopharma fermented molecules developed through advanced breeding and genomics*” (Richardson et al., 2019).

The biomedical industry too relies on computer systems and is equally vulnerable to cyber-attacks that may have national security implications. Cyber-biological capabilities could be targeted to disrupt disease surveillance systems, compromise medical response systems, or attack vaccine manufacturing supply chains (Dixon, 2021). As an example, in 2021 the US Bioeconomy Information Sharing and Analysis Center published findings regarding Tardigrade, a metamorphic, semi-autonomous advanced persistent threat (APT)^21^ that was detected in two US biomanufacturing facilities (Dotmatics 2022; Palmer et al., 2022; Reed and Dunaway, 2019 discuss the economic impact of the NotPetya ransomware attack of 2017, which resulted in a total direct cost to Merck (a pharmaceutical company) of almost $1 billion. For these reasons, Kitney et al. (2021) propose that vaccine productions needs to change to a more distributed model of manufacture for national resilience and preparedness (see Section 3.1).

##### 3.4.6.1 Mis-information, dis-information and propaganda

In addition to cyber-attacks, a cost-effective threat to national security can be achieved through the use of misinformation or disinformation (Palmer et al., 2021). An example provided by the authors involves the significance of the correct entries of data and findings in journals. It is possible that malicious actors may look to sabotage research to delay, cloud or stop progress and solutions in the future.

##### 3.4.6.2 Bio-terrorism

Without over-empathising the threat/capability, bioterrorism (a low-likelihood, high-impact threat that is relatively improbable but that could have a disproportionately large impact) has been identified as an accelerated concern due to biothreats that are now simpler and more accessible to terrorists (Townsend-Drake et al., 2021). For example, increased activity of the darknet to acquire, transfer or smuggle biological material or weapons and the use of drones by terrorist groups, have been identified by INTERPOL. Dixon (2021) provides a future potential example of malicious actors developing bioweapons that mimic the symptoms of common diseases in order to camouflage the initial spread of an engineered pathogen. Example accelerants highlighted in the report included emerging technologies, advances in biosciences, globalisation, the drivers of conflict and instability and vulnerabilities exposed by COVID-19. International security, according to Dixon (2021), continues to be challenged in an era of increased grey-zone conflict between great powers. Simultaneously, he suggests that advances in engineered biology are likely to enable novel capabilities and methods of plausibly deniable grey-zone manoeuvring.

### 3.5 Recommended solutions to the cyberbiosecurity problem in the literature

A new discipline–cyberbiosecurity–is recommended in 14 articles to address the sorts of security vulnerabilities discussed above (Mueller, 2021). Biosafety focuses on safety in the context of biological containment, such as microbial containment, which later progressed to genetically modified organism (GMO) safety issues such as releasing them in the open environment. Biosecurity refers to “*taking proactive measures to avoid intentional biohazards, such as the theft and misuse of biotechnology and microbiologically hazardous substances. It aims to reduce the risks associated with the misuse of synthetic biology which could cause harm to humans, animals, plants, or the environment through the creation, production, and deliberate or accidental release of infectious disease agents or their by-products (e.g., toxins)*” (Li et al., 2021). Neither addresses cyber risks. Consequently, cyber biosecurity (Richardson et al., 2019; Millet et al., 2019; Wintle et al., 2017; Dixon, 2021; Li et al., 2021) is defined as “*understanding the vulnerabilities to unwanted surveillance, intrusions, and malicious and harmful activities which can occur within or at the interfaces of comingled life and medical sciences, cyber, cyber-physical, supply chain and infrastructure systems, and developing and instituting measures to prevent, protect against, mitigate, investigate and attribute such threats as it pertains to security, competitiveness and resilience*” (Murch et al., 2018). According to Dixon (2021) “*cyberbio-security encompasses those biological, medical and genomic information security vulnerabilities that arise from the interfacing of living and non-living systems, and the integration of living (animate) and non-living (inanimate) information substrates*.” According to Logstail 2022 - Cyberbiosecurity aims to: “*understand the bio sciences specific risks and cyber threat landscape*.”

Millet et al. (2019) conducted an online survey with 13 professionals in the biotech industry and found that over ninety per cent (90%) of participants expressed a strong view that insufficient time and resources were being dedicated to dealing with these risks. Drape et al. (2021) conducted a virtual 2-day workshop and collected data from approximately 80 participants working in the food and agriculture sectors. They asked participants what they thought of cyberbiosecurity in their respective sectors and through qualitative analysis, found that Cyberbiosecurity is not a one size fits all solution (see Box 6 for unique considerations) and that it will need to be adapted for individual circumstances/applications (e.g., biomedical *versus* agriculture). Moreover, that to achieve this a common language among disciplines for professionals (a working lexicon) will be needed to help break language barriers for interdisciplinary collaboration. Further, according to Drape et al. (2021), training and resources for cyberbiosecurity should be available to businesses, companies, and other organisations to start investing in and improving their practices, noting that the lack of government involvement and programs may have prevented some from increasing their cyberbiosecurity practices.

BOX 6 **Five examples of unique considerations for various food and agriculture commodities from Duncan et al. (2019).**

1- Dairy: Gaps in the tracing of information and the potential for breaches of genetic data, herd health records and drug use which are regulated but for which data security is often limited.
2- Food animals: pedigree information of livestock breed (e.g., swine) could be manipulated leading to losses for producers.
3- Row crops: the challenge of traceability of grain production has led to enormous amounts of data (e.g., soil conditions, machinery location and performance). Aggregate data is often sent directly to a third parties for storage, cleaning, and processing. Although anonymization typically occurs at the time of aggregation, questions exist about the effectiveness of these techniques to protect against security vulnerabilities.
4- Fruits and vegetables: are the leading source of foodborne illness in the United States and tracking fresh produce from initial production through consumption is critical to limit the potential for, and impact of foodborne outbreaks.
5- Environmental resources (water): significant drinking water contamination incidents may be caused by cyberbio-attacks and cyber solutions are needed to ensure water safety on-farm, and for food processing to ensure consumer health and the proper functioning of the ecosystem.

Cyberbiosecurity solutions presented concerned connected laboratories and equipment (4 articles), digitised biological data and information sharing (16 articles), and organisational security measures and deterrence mechanisms (5 articles). These will be discussed in turn.

#### 3.5.1 Recommendations for connected laboratories and equipment

A connected laboratory is similar to a smart home in that its users’ can trigger physical changes to the environment (e.g., temperature, sound, motion) and receive notifications about such changes remotely. According to Reed and Dunaway, 2019, networked building automation systems and energy management software are commonly found in modern laboratory facilities, allowing for climate, pressure and humidity control between work-spaces to operate at varying levels of containment. These systems could be targeted by malicious actors. Murch et al. (2018) suggest that all cyber-physical interfaces should be secured including genome-editing, DNA assembly, synthesis and printing, portable genomic sequencers, AI for understanding biological complexity, autonomous systems and robotics in cloud labs, and, lab-on-a-chip and microfluidic technologies. According to Cunningham and Geis (2020), specific equipment that may be sensitive such as DNA synthesizers, should additionally be stored in secure access rooms. Gertner (2021) mentions that the newest high-containment labs have “air gapped” networks that are cut off from public internet traffic to prevent hacking.

#### 3.5.2 Recommendations for digitised biological data and material

For digitised biological data and material, Dieulis (2020) suggests that a risk assessment should be developed and applied on end-user intent. Mantle et al. (2019) suggest that there is a need for a dynamic cyber-biorisk assessment for manufacturing process control and product quality. To minimise the risk of malicious activity, Dieulis (2020) suggest that digital registries of bio-data track genetic designs via digital “signatures”. The authors further suggest inserting built-in constraints into design tools and implementing a DNA screening method enabled by ML (Farbiashand Puzis, 2020; Dieulis 2020; Jordan et al., 2020).

Several authors also point out that conventional cyber hygiene needs to be employed in the Life Sciences (Schneier and Larisa, 2019; Mueller, 2021; Wintle et al., 2017; Elgabry et al., 2020; Potter and Palmer, 2021; Reed and Dunaway, 2019; Mantle et al., 2019; Murch et al., 2018). Duncan et al. (2019) also report that a lack of basic cybersecurity training in agriculture is a problem. As a starting point, according to Reed and Dunaway, 2019, cybersecurity best practices should be implemented. According to Potter and Palmer 2021, networks of connected bioprocessing infrastructure require IT expertise for both management and security. Mueller (2021) identifies many web sites in the life sciences that provide methods for users to upload data that do not check for data integrity during the transfer process. Moreover, Wintle et al. (2017) suggest the standardization of biological information and methods for validating, storing and retrieving data by national agencies.

According to Mueller (2021) and Dixon (2021), IT solutions alone cannot deal with cyberbio concerns due to unrecognised convergence issues. Ibrahim et al. (2020) and Philp, 2021 suggest the use of DNA barcoding for traceability, and monitoring illegal activity and fraud (e.g., counterfeit products).

#### 3.5.3 Recommendations for information sharing

Opinions differed among the authors of the articles included in this review regarding information sharing. Schneier and Larisa (2019) suggest that both classified and unclassified information should be shared. In contrast, Cunningham and Geis (2020) suggest that for national security reasons, data and information must be kept under surveillance to prioritise threats and in so doing, controlling technology exports, while maintaining international cooperation to avoid ethical asymmetry. Cunningham and Geis (2020) suggest that Germany’s model of 50,000 € fine for biohacking (synthetic biology practiced outside the institutionalised and regulated premises) may be adaptable. Cunningham and Geis (2020) also suggest that horizon scanning with AI should be used by governments to monitor foreign investment, and ongoing academic research/grant proposals. Defranco et al., 2019 suggest that universities and research sites should record activities and according to Cunningham and Geis (2020) any company, university, or individual should make declarations and submit sequence information if conducting independent genetic work. According to Defranco et al., 2019, other information that should be shared publicly by companies and organisations include private and public recruitment of researchers, technology commercialisation, current/future technological military postures, and markets. Duncan et al., 2019 further suggest that suppliers of engineered biology data (and related products or services) must be approved.

#### 3.5.4 Recommendations for organizational security measures and deterrence mechanisms

Millet et al. (2019) suggest that staff and sponsored meetings should be used to implement what Peccoud et al. (2018) refers to as ‘blue-sky thinking’ to review workflows and identify cyberbiosecurity risks not covered by existing biosafety and biosecurity policies. Similarly, Elgabry et al. (2020) proposed the increased use of red-teaming (applying the hacker ethic of Information Technology in the Life sciences) as a way to move away from reactive changes (implemented after major events occur) to proactive governance in health security and biosecurity. Mueller (2021) suggests that it will be necessary to refine a list of cyberbiosecurity principles and goals to protect life sciences assets and that deterrence measures will need to consider emerging actors and their pathways of actions such as mechanisms for dual-use appropriation, ‘routes to harm,’ and multiple exposure pathways.

## 4 Discussion

Engineered biology is becoming more integrated within the cyber domain taking advantage of the benefits of internet connectivity yet the extent of its transformative impact on computing in the future remains a question. This SR explored the cyber implications of engineered biology as found in the literature, highlighting opportunities for cyber-biological crime prevention through public participation and more inclusive research studies, the need for introducing a crime risk assessment within the design and development of the internet-of-biological-things and more cyberbiosecurity solutions that can address the identified threats such as the development of LLM agent benchmarks relevant to the Life Sciences.

### 4.1 The need for the introduction of a crime risk assessment within the design and development of internet of biological things for regulatory bodies

Across these 52 articles, we identified a total of seven cyber opportunities including automated bio-foundries and the Internet-of-biological-things (IoBT). The combination of big datasets and artificial intelligence in bio-foundries enables streamlined and optimised biological design and manufacturing. The network of connected engineered biology in the form of biosensors and biological devices in IoBT distributed across geographies enables real-time and constant information exchange that can lead to precision agriculture with next-generation robotic architectures.

As we continue to develop these, a dynamic cyber-biological *crime* risk assessment for manufacturing process control and product quality may provide a mechanism towards “cyber-biosecurity by design” for devices and services. Current regulatory frameworks are limited to security risk assessments and guidance. These do not include *crime* risks and a more predictive approach could help fill this gap. Crime science, or the perspective of crime as an event that can be influenced directly by its immediate environment, may encourage the biotechnology industry to design-in security and crime out. This additional risk assessment is not proposed to slow down or hinder innovation but apply responsible security principles in the design and development lifecycle for clever criminocclusive design. As an observation, the language hard scientists use is different to a crime scientist. For example, the terms “hacking”, “penetration testing” or “red-teaming” biological sensing to intercept its reading, in the practice of science is often referred to as “testing the specificity” of a biological sensor. This is problematic because although both terms “hacking” and “specificity testing” in this case refer to the same meaning (the testing of the biological sensing capability), the former has an innate “think thief” (Ekblom, 2005) perspective that widens the anticipation landscape to include the active checking of other vulnerabilities. To illustrate this point, when conducting specificity testing on a biological sensing unit, it is cross-tested with similar analytes (that it is not intended to detect) as an activity of “intercepting” its reading. If activated, this would mean that biologically the sensor’s specificity requires optimization (one to one relation), while in the threat modelling landscape it would mean that it enabled multiple routes to hack the sensor (one to many relation). For example, a malicious actor could prepare a drink containing a molecule that interferes with the biological sensing unit to activate it for a false disease reading. Or the biosensor could be covered with that molecule, without the knowledge of the user of the device and so on. Although working on sensing specificity limits the sensor to recognize only one biomarker, it does not contribute to the ideation of effective and (inventive) crime prevention design that can be applied from the outset. A crime risk assessment therefore provides a distinct perspective from a safety or cybersecurity risk assessment that could future proof the design and development of emerging biotechnology such as the internet of biological things (Elgabry, 2023), particularly important as they continue to integrate across technologies and cyber-biological domains.

### 4.2 A call for cyberbiosecurity solutions and LLM agent benchmarking in the life sciences industry

A total of four main types of cyberbiosecurity solutions were identified in the literature. Briefly, for connected laboratories and equipment with cyber-physical interfaces (e.g., genome-editing, DNA assembly, synthesis and printing, portable genomic sequencers, AI for understanding biological complexity, autonomous systems and robotics in cloud labs, and, lab-on-a-chip and microfluidic technologies), it is recommended that they are better secured. Digital entries, signatures and an end-user intent risk assessment is needed for securing digitised biological data and material. That there is a need for more oversight on information sharing and finally, the implementation of red-teaming to organisational security measures an deterrence mechanisms. The SR indicates that there is a need for more solutions for cyber-biosecurity, this can include DNA cryptography (Berezin et al., 2024) and use of known cybersecurity methods onto biology such as Data mining (Shankar et al., 2023). Data mining is the process of extracting useful patterns, information, and expertise from massive datasets; a promising avenue for further investigation as a means of mitigating cyber-biological attacks.

This SR identified a total of four cyber threats such as Artificial Intelligence misuse and biological dataset targeting. The increasing use of large language models (LLMs) such as Chat-GPT, has extraordinary implications in positively increasing productivity and creative applications (Al Naqbi et al., 2024) but may also have some misuse implications that we would need to be particularly weary of. For example, MIT students use LLM chatbots to design a pandemic pathogen in 1 h, 4 potential pandemic pathogens suggested that DNA synthesis companies unlikely to screen (Soice et al., 2023). Further implications of LLM *agents* - enhancing the single-step generation to multiple LLMs and tools for accomplishing complex multi-step tasks - that could misuse or automate even parts of biotechnology workflow. For example, as did the AI- drug discovery pharmaceutical company that inverted their in-hour AI-powered drug discovery algorithm, resulting to the *de novo* design of 40,000 potential biochemical weapons in under 6 h (Urbina et al., 2022). Therefore, even more worrisome would be an LLM agent that was able to perform a cyber-biological attack. For example, Farbiash and Pusiz (2020) showed that a DNA injection attack is possible that could obfuscate a sequence of concern (SoC) order from a DNA synthesis order. Tasks of concern for an LLM agent would include the successful implementation of the SoCO2 algorithm described to produce an obfuscated sequence, such that BLAST indicates greater alignment with the camouflage genes than the sequence of concern. Therefore developing a benchmark that evaluates LLM agents' abilities to contribute to the replication and extension of cyberbiosecurity research is pivotal. While there have been efforts to assess the risk that a large language model (LLM) could aid someone in creating a biological threat, such as OpenAI’s recent publication^22^, the launch of new features and versions such as GPT-4o at the time of writing paper requires iterative attention that could be facilitated through mechanisms such as the BAKE framework (Elgabry, 2023). In fact there are call for projects that allocate substantial funds to developing a benchmark for LLM agents^23^.

### 4.3 Cyber-biological crime prevention through public participation and more inclusive research studies

Among the identified threats in this review, such as bio-discrimination and misinformation/disinformation, another crucial aspect of cyber-biosecurity that warrants attention is the need for public participation and more inclusive research studies. Notably, a significant amount of research is conducted predominantly on males, potentially leading to vulnerabilities in less understood genders. This gender bias in research may result in treatments being less effective or understood for women’s bodies, raising the question: What does biosecurity for women entail?

Additionally, public participation as a biosecurity strategy should be prioritized. Lessons from the COVID-19 pandemic have shown that measures were more effective when the public had a better understanding (Fridman et al., 2020). A biosurveillance platform that incorporates public participation and allows for opt-in could provide a valuable mechanism. This approach not only enhances biosecurity but also calls for broader community involvement (Elgabry, 2023).

As Engineered biology is becoming more integrated within the cyber domain to take advantage of the benefits of internet connectivity and the transformative impact on computing in the future, a total of 9 policy recommendations that can be utilized by various entities, including governments, are provided in the next section to ensure that cyberbiosecurity remains frontline in a growing and thriving bioeconomy.

## 5 Limitations

• Must acknowledge that the review only captures open data and that any classified information that may be relevant but sensitive to national security is not included.
• Moreover that only English articles are extracted does not allow an exhaustive reflection of other nations’ progress in the engineered biology industry. For example, in 2010 China published a national Science and Technology strategy with a roadmap to 2050 that heavily focused on biotechnology, but was not included in this review (Strategic General Report of the Chinese Academy of Sciences, 2010).

## 6 Policy recommendations for governments

1. Cyber opportunities for the new era of engineered biology need a data infrastructure that can support it with standardised data exchange formats, data management and curation methods, metadata reporting, and data interoperability using open-source software.
2. Cyber threats continue to effect small and large businesses due to the lack of cyber hygiene practiced. This suggests a need for the enforced adoption of minimum cyber standards such as through the NCSC cyber essentials, https://www.ncsc.gov.uk/cyberessentials/overview.
3. Training and resources for cyberbiosecurity should be available to businesses, companies, and other organisations to start investing in and improving their practices, noting that the lack of government involvement and programs may have prevented some from increasing their cyberbiosecurity practices.
4. Designing adversary-resilient biological protocols are critically needed as standard end-to-end encryption provided by HTTPS does not help when the data is corrupted from, for example, a malicious browser plugin or emerging cyberbiorisks.
5. Gene libraries most commonly used (e.g., GeneBank, NCBI) should provide digital signatures for data downloaded and require validation from requested orders, to enable intrusion detection approaches and to identify malicious code.
6. Critical infrastructures such as vaccine production need to change to a more distributed model of manufacture to create more resilience in the system.
7. Cyberbiosecurity is not a one size fits all solution (see Box 6 for unique considerations) and will need to be adapted for individual circumstances/applications (e.g., biomedical *versus* agriculture). To achieve this a common language among disciplines for professionals (a working lexicon) will be needed to help break the language barriers that occur in interdisciplinary collaboration. Additionally, ‘blue-sky thinking’ will be required to review workflows and identify cyberbiosecurity risks not covered by existing biosafety and biosecurity policies. The use of “red-teaming” (applying the hacker ethic of Information Technology in the Life sciences) may offer a solution that moves away from reactive changes (implemented after major events occur) to proactive governance in health security and biosecurity.
8. For digitised biological data and material, a risk assessment mechanism should be developed and applied on end-user intent.
9. Biotechnology literacy is needed to improve the public’s perception of biotechnology

## 7 Conclusion

Engineered biology has the potential to positively transform future society through many application areas including health, sustainability and agriculture. The impact of engineered biology in cyberspace is pivotal. This systematic review analysed 52 articles and addressed the main cyber opportunities and/or threats related to engineered biology, as well as how quickly engineered biology is likely to evolve in the next 5–10 years. A total of seven cyber opportunities including automated bio-foundries and four cyber threats such as Artificial Intelligence misuse and biological dataset targeting were identified. Nine policy recommendations that can be utilized by various entities, including governments, were provided that may address the cyber threats identified.

The findings of this review were discussed, highlighting the need for cyber-biological crime prevention through public participation and more inclusive research studies as well as the introduction of a crime risk assessment within the design and development of internet of biological things. A call for cyberbiosecurity solutions and LLM agent benchmarking in the Life Sciences was also emphasised as engineered biology continues to converge with other emerging technologies and cyberspace.

## Data Availability

The raw data supporting the conclusions of this article will be made available by the authors, without undue reservation.

## Footnotes

1 DSTL, 2022 https://www.gov.uk/government/publications/engineering-biology-for-defence-and-security/competition-document-engineering-biology-for-defence-and-security#:∼:text=Introduction,world%20solutions%20is%20engineering%20biology.

2 ProQuest Central is a comprehensive search engine for academic literature (it covers 63 Databases across all major subject areas, including business, health and medical, social sciences, science, and technology. It includes full-text scholarly journals, Newspapers, magazines, Dissertations, working papers, case studies, and Market reports. These include the Criminal Justice Database, Computing Database, Library Science Database, Science Database, Social Science Database, Psychology Database and continent-specific databases covering technology and social sciences (such as the Australia and New Zealand Database, Continental Europe Database, East and South Asia Database, East Europe and Central Europe database, etc.), ProQuest Dissertations and Theses Global), it also includes the Association for Computing Machinery (ACM) digital library).

3 ACM digital library is a comprehensive database of full-text articles and bibliographic literature covering computing and information technology from Association for Computing Machinery publications.

4 IEEE Xplore Digital Library is an indexed database of articles and papers on computer science, electrical engineering and electronics from the Institute of Electrical and Electronics Engineers (IEEE) and the Institution of Engineering and Technology.

5 The search query provided was applied to the ProQuest Central database. Variants of this was applied to the other databases searched.

6 An evidence-based minimum set of items (27-item checklist and a 4-phase flow diagram) for reporting in systematic reviews and meta-analyses to facilitate and demonstrate preparation and reporting of a robust protocol for a systematic review (Moher et al., 2015).

7 Inter-rater reliability (IRR) was assessed based on two coding categories (i.e., inclusion *versus* exclusion) using the prevalence- and bias-adjusted kappa (PABAK) statistic, which controls for chance agreement. For this review, the PABAK score of 1.0 at the Title/Abstract stage indicated perfect agreement between the two reviewers.

8 NBCC (2016) Chapter 12 Process Development - Biomanufacturing. Northeast Biomanufacturing Center and Collaborative, Last Accessed here: https://biomanufacturing.org/uploads/files/701306457399630057-chapter-12.pdf.

9 The sharing, leasing, reusing, repairing, refurbishing and recycling existing materials and products for as long as possible as a model of production and consumption.

11 The characterization and quantification of collective pools of (Big) data of biological molecules (e.g., genomics, transcriptomics, proteomics, metabolomics, and metagenomics) that translate into the structure, function, and dynamics of an organism or organisms (Lay et al., 2006).

12 A technology that allows transparent information sharing on a network that typically uses cryptography to verify and secure transactions.

13 Strain engineering refers to the use of synthetic biology techniques to create and optimize microbial strains that produce target proteins. Automation accelerates these processes and enables parallel strain engineering.

14 Now (2022) acquired by Ginkgo Bioworks, a cell-programming company that uses genetic engineering to produce bacteria with industrial applications.

15 Vogue Business (2022) https://www.voguebusiness.com/sustainability/balenciaga-releases-coat-made-with-ephea-a-leather-alternative Accessed on 18/12/2022.

16 Electronic circuits that mimic neuro-biological architectures (Schuman et al., 2022).

17 Two communicating parties are intercepted by an attacker positioned between them to alter data traveling between them without their knowledge.

18 The use of database code (SQL) for backend database manipulation to access information that was not intended to be displayed.

19 The study of how behaviours and the environment can cause changes that affect the way genes work.

20 The ecosystem of microbes that reside in the gastrointestinal tract which are responsible for various functions such as host nutrient metabolism, drug metabolism, immune-modulation, and protection against pathogens.

22 Building an Early Warning System for LLM-Aided Biological Threat Creation, OpenAI, 31 January 2024, openai.com/index/building-an-early-warning-system-for-llm-aided-biological-threat-creation.

23 “Request for Proposals: Benchmarking LLM Agents on Consequential Real-World Tasks.” Open Philanthropy, 4 May 2024, www.openphilanthropy.org/rfp-llm-benchmarks/.
